# Dal Pont vs Hunsuck: Which Technique Can Lead to a Lower Incidence of Bad Split during Bilateral Sagittal Split Osteotomy? A Triple-blind Randomized Clinical Trial

**DOI:** 10.29252/wjps.10.3.25

**Published:** 2021-09

**Authors:** Farhad Zeynalzadeh, Zahra Shooshtari, Majid Eshghpour, Seied Hosein Hoseini Zarch, Elahe Tohidi, Sahand Samieirad

**Affiliations:** 1 Oral and Maxillofacial Department, Mashhad University of Medical Sciences, Mashhad, Iran; 2 Student Research Committee, Mashhad University of Medical Sciences, Mashhad, Iran; 3 Oral and Maxillofacial Diseases Research Center, Mashhad University of Medical Sciences, Mashhad, Iran.; 4 Oral and Maxillofacial Radiology, Oral and Maxillofacial Diseases Research Center, Mashhad University of Medical Sciences, Mashhad, Iran.; 5 Dental Research Center, Mashhad University of Medical Sciences, Mashhad, Iran.

**Keywords:** BSSO, Bad split, Dal pont osteotomy, Hunsuck osteotomy

## Abstract

**BACKGROUND:**

We aimed to assess the incidence of bad split fractures during Bilateral Sagittal Split Osteotomy (BSSO) mandibular setback surgery using Dal Pont and Hunsuck techniques.

**METHODS:**

All healthy adults with skeletal class III discrepancy, who were candidates for mandibular setback surgery were enrolled in this randomized clinical trial in the Maxillofacial Surgery Department of Qaem Hospital, Mashhad, Iran; from 2018-2020. These patients were randomly divided into two equal groups; one group underwent BSSO using Dal Pont osteotomy while the Hunsuck osteotomy was employed for the other group. A bad split fracture which identified through intra-operative clinical and postoperative radiographic examination was the outcome variable. The significance level was set at 0.05 using SPSS 16.

**RESULTS:**

Overall, 104 consecutive patients, comprising of 52 (50%) males with an average age of 23.09±3.08 were recruited. The average duration of osteotomy and splitting was reported to be 22.74±3.06 min. 10 bad split fractures (9.62%) were observed; 7 of which occurred in the Dal Pont group and 3 in the Hunsuck group. However, this difference was not significant. In 80% of the cases, bad split osteotomy occurred in the proximal segment, while this finding was identified in the distal segment in 20% of cases. The average duration of osteotomy and splitting was significantly longer in the Dal Pont group (P<0.001).

**CONCLUSION:**

The duration of osteotomy and splitting is much shorter when the Hunsuck technique is employed, and the incidence of unfavorable fractures is also less compared to the Dal Pont osteotomy technique.

## INTRODUCTION

Orthognathic surgeries are considered to be the mainstay of treatment for correcting severe jaw discrepancies and malocclusions^1^^-^^3^. Skeletal class 3 deformities that require surgical intervention seem to be quite frequent among the Iranian population^1^^, ^^4^.

Bilateral Sagittal Split Osteotomy (BSSO) is a well-known and highly favored technique for correcting mandible growth deformities^1^^, ^^3^^-^^8^. This technique was initially introduced by Obwegezer and multiple modifications have been proposed ever since. Dal Pont, Hunsuck, Trauner and Epker modifications are acclaimed BSSO modified techniques in orthognathic surgery^1^^, ^^3^^-^^12^. 

Despite these profound modifications, troublesome complications such as neurosensory disturbances, excessive bleeding, and unfavorable fractures still remain a problem while performing BSSO surgery^1^^-^^5^^, ^^13^^, ^^14^. Unfavorable and unanticipated sagittal split osteotomies are referred to as bad split fractures. These fractures can cause unstable surgical fixation, malunion, and infection in the osteotomy site^1^^-^^5^^, ^^12^^-^^14^. 

The incidence of unfavorable bad split fractures varies from 1% to 23 % in previously conducted studies^1^^, ^^4^^, ^^6^^, ^^7^^, ^^12^^, ^^15^^-^^17^. The patient’s age, gender, mandibular anatomy, presence of mandibular third molar at surgery as well as the surgeon’s expertise are all contributing factors to the occurrence of bad split fractures^1^^, ^^4^^, ^^6^^, ^^7^^, ^^12^^, ^^15^^-^^18^. 

Dal Pont and Hunsuck modifications are both well-established procedures in mandibular setback BSSO surgery, and somewhat similar^3^^, ^^6^^, ^^7^^, ^^12^. Both techniques require a lateral osteotomy cut between the first and second molar, while the medial osteotomy cut is what differs between these two techniques^3^^, ^^6^^, ^^7^^, ^^12^. In the Hunsuck technique, the medial cut ultimately extends to the lingula in the medial aspect of the lingual surface of the ramus (short-cut medial osteotomy), while the medial cut in the Dal Pont technique reaches the posterior border of the ramus (long-cut medial osteotomy)^3^^, ^^6^^, ^^7^^, ^^12^.

The incidence of bad split fractures would be the same after Dal Pont and Hunsuck osteotomy. Based on our previous literature review, no study has to date exclusively compared the incidence of bad split fractures after Dal Pont and Hunsuck osteotomy in the absence of the mandibular third molars^1^^-^^4^^, ^^6^^, ^^7^^, ^^12^^, ^^14^^, ^^19^. 

Hence, we aimed to investigate the incidence of bad split fractures after Dal Pont and Hunsunk osteotomy through a triple-blind randomized clinical trial. 

## METHODS AND MATERIALS

The protocol of this randomized clinical trial was approved by the Research and Ethics Committee of Mashhad University of Medical Sciences (IR.MUMS.DENTISTRY.REC.1398.008) and was registered in IRCT under the code IRCT20150613022697N7. Guidelines of the declaration of Helsinki were followed in this research. Patients were only recruited after obtaining fully informed written consent.

Healthy adults with an American Society of Anesthesiologists (ASA) status I or II, between the ages 18 and 40 were included in this study. Participating patients had skeletal class III deformity with mandibular prognathism and were candidates for mandibular setback surgery through BSSO (Bilateral Sagittal Split Osteotomy) in the Maxillofacial Surgery Department of Qaem Hospital, Mashhad, Iran; from the years 2018-2020.

All patients had their mandibular third molars removed, at least 6 months prior to orthognathic surgery. Atypical mandibular anatomy for instance an extremely thin mandibular cortex or close proximity between the mandibular lingula and sigmoid notch was identified through preoperative radiographic examination; these cases were excluded from the study. Patients with a history of maxillofacial trauma or previous maxillofacial surgery, developmental disorders affecting the jaws, craniofacial syndromes, and alveolar clefts were all excluded from the study. Patients refusing to complete the postoperative follow-up visits were also omitted from the study.

Consecutive patients were randomly allocated and divided into two equal groups; the Dal Pont group and the Hunsuck group. This was accomplished through the block randomization technique. Allocation concealment was performed using sequentially opaque sealed envelopes. All the packages were labeled and numbered randomly, and the codes were kept in a secure location until the end of the study. Although the randomization codes were concealed from the patient, data analyzer, radiologist, and student (triple-blind randomized clinical trial); the treating surgeon and anesthesiologist were completely aware of which group the patient had been assigned to.

**Fig. 1:**
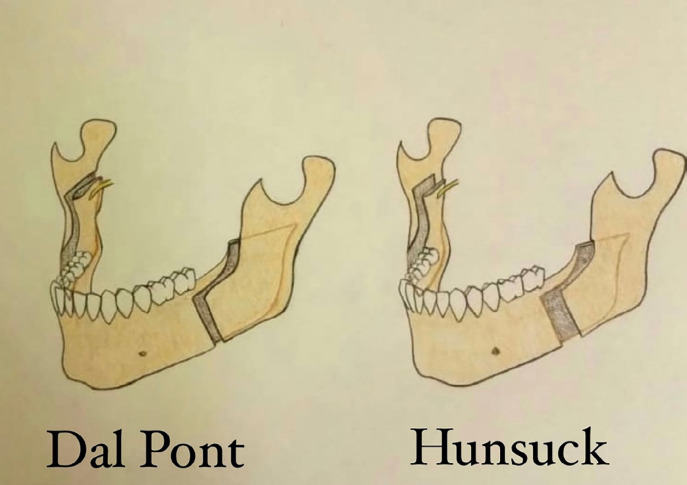
The schematic view of the Dal Pont and Hunsuck techniques

All cases underwent mandibular setback surgery through BSSO and were treated by the same surgical team. Depending on the group the patient was assigned to, either Dal Pont or Hunsuck technique was employed. The mentioned techniques are both well-established procedures in orthognathic surgery, and somewhat similar. Both techniques require a lateral (vertical) cut between the first and second molar, while the medial cut is what differs between these two techniques. In the Hunsuck technique, the medial cut extends to the lingula in the medial aspect of the lingual surface of the ramus (short-cut medial osteotomy), while the medial cut in the Dal Pont technique reaches the posterior border of the ramus (long-cut medial osteotomy) (Figure 1).

An unfavorable split was identified through intraoperative clinical examination and postoperative radiographic examination. The patient’s panoramic and posterior-anterior cephalometric radiographs were carefully analyzed by an oral and maxillofacial radiologist, in order to detect any possible bad splits in the osteotomy region, the distal or the proximal osteotomy segment; coronoid process or condylar region.

The patient’s age, gender, employed surgical technique (Dal Pont or Hunsuck), duration of the surgical procedure, the occurrence of bad split osteotomy and the exact location of the unfavorable split; were all recorded in a corresponding checklist. 

The type of sagittal split osteotomy, either Dal Pont or Hunsuck modification, was the study predictive variable. The incidence of bad split osteotomy during BSSO mandibular setback surgery was the outcome variable.

The randomization codes remained concealed from the radiologist and the data analyzer until the end of the study.

All data were collected and sent for statistical analysis using SPSS (ver.16, Inc, Chicago, IL). Chi-square test, Mann-Whitney, Independent T-test, and Fisher’s exact test were incorporated in the statistical analysis process. As for descriptive analysis, appropriate charts and tables were used to display the central tendency and dispersion indexes. *P*-values less than 0.05 were considered statistically significant.

## RESULTS

Overall, 104 consecutive patients, comprising 52 (50%) males and 52 (50%) females, with an average age of 23.09±3.08 yr and an age range of 18 to 40 yr were enrolled in this randomized clinical trial. Among all the performed operations, bad split osteotomy was only identified in 10 cases (9.62%); 7 (70%) of which occurred in the Dal Pont group and 3 (30%) in the Hunsuck group.

**Fig. 2:**
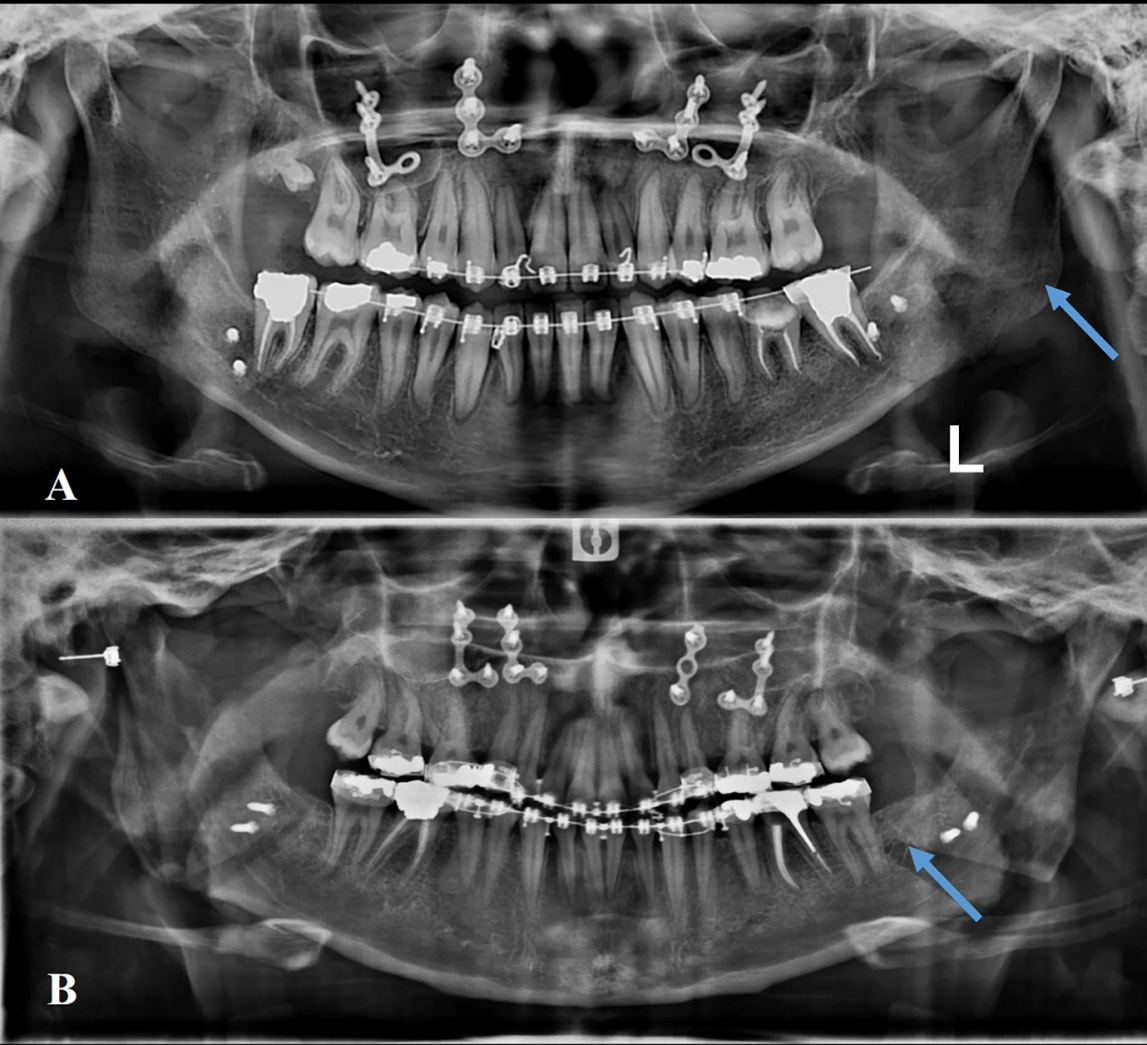
The radiographic view of bad split in the Hunsuck (A) and Dal Pont (b) techniques

Out of the 10 bad splits, 7 (70%) of them were related to the left side of the mandible and the other 3 (30%) occurred in the right side. In 8 (80%) of the cases, bad split osteotomy occurred in the proximal segment, while this finding was identified in the distal segment in 2 (20%) cases. All the identified unfavorable splits were detected through intraoperative clinical examination as well as postoperative radiographs (Figure 2).

The average duration of the surgical procedure (BSSO) was reported to be 22.74±3.06 min; the longest and shortest operation time was 15 and 31 min, respectively.

Independent t-test revealed that although the average age of the patients in the Hunsuck group (23.26±2.99 yr) was slightly higher than the Dal Pont group (22.92±3.19 yr). However, this difference was not statistically significant (*P*=0.584).

According to the Chi-square test, patient distribution frequency was not statistically different between the two treatment groups (*P*=1.00). The Dal Pont and Hunsuck group both consisted of 26 (50%) males and 26(50%) females.

The amount of mandibular displacement ranged from 2 to 5mm in both groups, and no significant difference was noted concerning this factor (*P*=0.745).

Table 1 depicts the duration of the BSSO procedure between the two groups. The time spent for osteotomy and splitting ranged from 15 to 27 min in the Hunsuck group and was 2.18±2.65 min long on average, while this procedure took between 20 to 31 min in the Dal Pont group with an average time of 24.30±2.64 minutes. Based on the independent t-test, the average duration of osteotomy and splitting was significantly longer in the Dal Pont group (*P*<0.001).

As displayed in Table 2, the number of bad split osteotomies in the Dal Pont and Hunsuck group was reported to be 7 and 3, respectively. Although the incidence of unfavorable fractures during BSSO was higher in the Dal Pont group, this difference was not proven to be statistically significant (*P*=0.182).

Out of the 7 bad split osteotomies identified in the Dal Pont group; 4 (57.1%) occurred in the left ramus while the other 3(43.9%) were related to the right side of the mandible. On the contrary, all 3(100%) bad split osteotomies which occurred in the Hunsuck group, happened to be in the left ramus. Despite the fact that the incidence of unfavorable fractures was more frequent in the left mandible compared to the right mandible; this difference was not considered statistically significant (*P*=0.475). Table 3 presented this matter in further detail as well as Figure 2 which illustrates bad split fractures in the left mandible using both Dal Pont and Hunsuck techniques (Figure 2).

Buccal cortical plate fractures (proximal segment) were seen after Dal Pont and Hunsuck osteotomy in 6 and 2 cases, respectively. While only one case of lingual plate fractures (distal segment) was reported in each of the two groups. The incidence of unfavorable fractures was higher in the proximal segment when compared to the distal segment; but this difference was statistically insignificant (*P*=0.300) (Table 4).

In the Hunsuck group, bad split fractures occurred in 2 females and 1 male patient, this difference was not significant. Regarding the Dal Pont group, 4 females and 3 males experienced bad splits during their surgical procedure; but this difference was again not statistically significant (*P*>0.99). In total, the bad split fractures were observed in 6 females and 4 males, but this difference was still not considered statistically significant. (Table 5)

No cases of coronoid or condylar neck fracture were present what so ever.

**Table 1:** The comparison of Osteotomy and Splitting Duration in Hunsuck and Dal Pont Groups

Group	Number	Average	Standard Deviation	Minimum	Maximum	Median	Independent t-test results
Hunsuck	52	21.18	2.65	15	27	21.0	t=5.90 *P*<0.001
Dal Pont	52	24.30	2.64	20	31	24.0	

**Table 2:** The incidence of Bad Split Fractures in the Hunsuck and Dal Pont Group

		Identified Bad Split		Total
		No	Yes	
Group	Hunsuck	49	3	52
		94.23	5.77	100.0
	Dal Pont	45	7	52
		86.54	13.46	100.0
Total		94	10	104
		90.38	9.62	100.0
Chi-square Test results		X^2^=1.78 *P*=0.182		

**Table 3:** The distribution of Bad Split Fractures in the Right and Left side of the Mandible

		Ramus		Total Bad Split Fractures
		Right	Left	
Group	Hunsuck	0	3	3
		0.0%	100.0%	100.0%
	Dal Pont	3	4	7
		42.9%	57.1%	100.0%
Total		3	7	10
		30.0%	70.0%	100.0%
Fisher's Exact Test results		P=0.475		

**Table 4:** The Distribution of Bad Split Fractures in the Proximal and Distal Segments

		Segment		Total	Fisher's Exact Test results
		Proximal	Distal		
Group	Hunsuck	2	1	3	*P*=0.300
		66.7	33.3	100.0	
	Dal Pont	6	1	7	
		85.72	14.28	100.0	
Total		8	2	10	
		80.0	20.0	100.0	

**Table 5:** The distribution of Bad Split Fractures among Male and Female Patients in both Study Groups

Group	Gender	Identified Bad Split		Total	Fisher's Exact Test results
		No	Yes		
Hunsuck	Female	24	2	26	*P*>0.99
		92.30	7.70	100.0	
	Male	25	1	26	
		96.15	3.85	100.0	
	Total	49	3	52	
		94. 23	5.74	100.0	
Dal Pont	Female	22	4	26	*P*>0.99
		84.61	15.39	100.0	
	Male	23	3	26	
		88.46	11.54	100.0	
	Total	45	7	52	
		86.54	13.46	100.0	
Total	Female	46	6	52	χ2=0.44 P*=0.505
		88.46	11.54	100.0	
	Male	48	4	52	
		92.3	7.7	100.0	
	Total	90	10	100	
		90.0	10.0	100.0	


## DISCUSSION

The present study was a triple-blind randomized clinical trial, in which the incidence of bad split osteotomy during BSSO surgery through Dal Pont technique (long-cut medial ramus osteotomy) and Hunsuck technique (short-cut medial ramus osteotomy) was compared and investigated in patients who had their third molars removed prior to orthognathic surgery.

 The two mentioned techniques are widely performed modifications of the traditional Obwegezer SSRO procedure which was first introduced to the field of surgery in 1957^9^^, ^^10^^, ^^12^^, ^^20^. Bilateral Sagittal Split Osteotomy is to date the most favorable osteotomy technique for correcting mandibular prognathism, retrognathism, and asymmetry^6^^, ^^7^^, ^^9^^, ^^10^^, ^^12^^, ^^20^. 

Multiple complications have been associated with this surgery (BSSO), such as edema, neurosensory disturbances, temporo-mandibular joint dysfunction, condylar resorption, and displacement, as well as bad split fractures, extensive bleeding, infection, and skeletal relapse^2^^, ^^5^^-^^8^^, ^^13^^, ^^14^^, ^^19^^, ^^21^^-^^24^. In order to abate the mentioned complications, several modifications for BSSO surgery have been advocated over the years^1^^, ^^6^^-^^8^^, ^^12^^, ^^17^^, ^^25^. 

Unfavorable bony splits are a commonly encountered complication of BSSO surgeries, with a reported incidence of up to 23%^2^^, ^^4^^, ^^6^^-^^8^^, ^^12^. The occurrence of unfavorable splits is genuinely possible after both Hunsuck and Dal Pont modifications^2^^, ^^4^^-^^8^^, ^^12^^,^^14^; hence determining and choosing a technique with minimum risks for such a problem can definitely lead to a much more satisfactory surgical procedure ^2^^-^^8^^, ^^12^^, ^^14^^, ^^18^^, ^^25^. Bone sequestration, delayed or malunion of the bony segments and infection are potential consequences of unfavorable fractures ^2^^, ^^5^^-^^8^^, ^^13^^, ^^19^^, ^^23^^-^^26^. Bad split fractures can occur in the tooth-bearing distal segment as well as the proximal segment which includes the condyle and coronoid process. Being aware of the possible etiologic factors can aid in reducing the chances of unfavorable fractures^1^^, ^^3^^, ^^8^^, ^^17^^, ^^23^^-^^26^.

The incidence of bad split osteotomies in the present study was reported to be 9.62% in total, and although bad splits were more frequently observed after Dal Pont osteotomy compared to Hunsuck osteotomy; however, this difference was not statistically significant. The sample size of our study (104 patients in total and 52 in each group) was enough and more than the number of consecutive patients in other studies ^1^^, ^^3^^, ^^24^. 

Dal Pont has proposed a pattern that provides a larger common surface between the split bone segments and yields bone integrity; this modification made the further displacement of the distal segment feasible ^6^^, ^^7^^, ^^12^^, ^^20^. The Hunsuck and Epker modification entails an incomplete lingual osteotomy which ends just behind the lingula and inferior alveolar neurovascular bundle (IANB). This modification is considerably easier compared to the conventional lingual osteotomy which would traditionally extend to the posterior border of the ramus and occasionally cause bad split fractures^6^^, ^^7^^, ^^9^^, ^^10^^, ^^12^^, ^^27^. 

The fracture patterns were investigated following Obwegezer/Dal Pont and Hunsuck Epker modifications of BSSO^13^. Overall, 124 postoperative CBCTs were evaluated and 60 splits were analyzed through these techniques. The incidence of bad split fractures was reported to be 11.3% after Dal Pont osteotomy and 10% after Hunsuck osteotomy^13^. A significant correlation between the employed technique and the incidence of unfavorable fractures was established in the above-mentioned study^13^; while a higher incidence rate after Dal Pont osteotomy compared to Hunsuck osteotomy was also in line with the findings of our study.

Zamiri et.al have also assessed and compared the fracture patterns in the medial cortex after medial long-cut versus medial short-cut techniques^3^. Three different fracture patterns following BSSO surgery were observed, but the type of medial cut and the ensuing fracture pattern were not significantly correlated. Therefore, the length of the medial cut does not affect the incidence of bad split fractures^3^. On the other hand, the thickness of the ramus was recognized as an influential factor in the incidence of unfavorable fractures^3^. Complicated and atypical mandibular anatomy, inappropriate osteotomy patterns along with the presence of impacted third molars are predisposing factors for unfavorable fractures^1^^, ^^3^^, ^^4^^, ^^6^^, ^^7^^, ^^18^^, ^^21^^, ^^24^. The ascending ramus can demonstrate various types of anatomy; the ramus thickness can also differ from patient to patient. The absence of cancellous bone between the two surrounding cortical plates precludes simple osteotomy splitting^1^^, ^^3^^, ^^6^^-^^8^^, ^^17^^, ^^24^. 

The patient’s age, presence of impacted third molar at surgery, incomplete osteotomy of the inferior border of the mandible, the surgeon’s expertise, and the mandibular anatomy; can all potentially contribute to the occurrence of unfavorable fractures^6^. A systematic review was devoted to evaluating the incidence of bad split fractures after BSSO surgery. Overall, 458 bad splits were identified among 19527 BSSO surgeries in 10271 patients and the overall incidence of bad splits was reported to be 2.3% of all sagittal split osteotomies. The most commonly encountered unfavorable fractures were buccal plate fractures of the proximal segment and lingual plate fractures of the distal segment^6^. 

The incidence of bad split fractures after Dal Pont or long-cut medial ramus osteotomy was almost 4 times greater than that after Hunsuck or short-cut medial ramus osteotomy. The present study also revealed a higher incidence of bad split fractures after Dal Pont osteotomy compared to Hunsunk Osteotomy^6^, which was consistent with our study findings. Females, older patients, and those with mandibular third molars were more susceptible to bad split fractures^6^^, ^^7^. When the operation was performed by a right-handed surgeon, bad split fractures were more likely to occur in the left mandible and they were more frequent in the proximal segment compared to the distal segment^6^^, ^^7^. These findings were in accordance with the results of our study. 

The presence of impacted third molars at orthognathic surgery has been related to unanticipated fractures during BSSO surgery and these teeth can jeopardize the mandible’s integrity and cause bad split fractures in the proximal or distal segments^1^^, ^^4^^, ^^6^^-^^8^^, ^^15^^-^^17^^, ^^23^. This matter still remains a subject of controversy^4^^, ^^15^^, ^^16^. Some surgeons advocate third molar removal at least 6 months before surgery, while others claim that simultaneous removal of an impacted third molar during BSSO does not seem to contribute to bad split fractures^1^^, ^^4^^, ^^6^^-^^8^^, ^^15^^-^^17^^, ^^23^.

A study was conducted the effect of mandibular third molars on the incidence of bad split osteotomies during BSSO surgery in 140 skeletal class 3 patients^4^. The incidence of bad split fractures was 3.7 greater in patients with mandibular third molars, compared to those who had their third molars removed prior to surgery. The chances of unfavorable fractures were 1.7 times higher in females compared to male patients. Moreover, on that account, highly advocated the third molar removal at least 6 months before orthognathic surgery^4^. In our study females were also more prone to bad split fractures similar to previous study ^4^. Moreover, the mandibular third molars had been removed prior to surgery in all cases to eliminate the confounding factors.

The presence of mandibular third molars is the only predictive factor for the occurrence of bad split fractures ^19^. On the contrary, the patient’s age, gender, occlusion, and the surgeon’s expertise were not significantly related to the occurrence of bad split fractures^19^. This is exactly why third molar removal was obligatory before BSSO surgery in this study. 

The incidence of bad split fractures and subsequent primary healing of the mandible was scrutinized^5^. Overall, 262 patients were subjected to BSSO surgery and 524 sagittal split osteotomies were evaluated. The presence of third molars at surgery was not correlated to the incidence of bad split fractures; while females, patients aged over 40 yr old and an obtuse Gonion angle (90-115 degrees) were significantly related to the incidence of unfavorable fractures^5^. The effect of mandibular anatomy parameters was investigated on the occurrence of bad fractures during BSSO^24^. The patients’ postoperative CBCTs were analyzed. Patients with a shorter ramus and a low thickness of the buccolingual alveolar region distal the second molar had a higher risk of unfavorable fractures^24^. Taking this into account, patients with craniofacial syndromes and those presenting severely deformed mandibles were excluded from our study. 

Older age is definitely correlated to a higher risk of bad split fractures during SSRO^2^^, ^^6^^, ^^7^^, ^^14^. The relationship between the occurrence of a bad split and mandibular anatomy upon SSRO was evaluated^1^. The buccolingual thickness of the retromandibular area, the buccolingual of the ramus at the level of the lingula, the height of the mandible from the alveolar crest to the inferior border of the mandible, and the anterior-posterior width of the ramus were measured through CBCTs. Mandibular anatomy can significantly affect the risk for bad split fractures when a BSSO surgery is being performed ^1^. A thin retromolar area can increase the risk for bad split osteotomies in the buccal or lingual plate of the proximal or distal segments. This may be attributable to the fact that a fragile and inadequate mandibular bone cannot tolerate usual osteotomy forces and are more prone to unfavorable fractures^1^. 

It is important for the oral and maxillofacial surgeon to aware of the associated risk factors for unfavorable fractures in order to perform an optimal surgical procedure with minimal complications. 

***Suggestions and Limitations***

While the results of this randomized clinical trial are encouraging, they are not without limitations. Since this study was carried out through a small population, it would be best if similar studies with a multicenter population were conducted across the country. Blinding the surgeon was obviously not possible; this is also considered a shortcoming of this study. It would also be beneficial if future studies investigate the relationship between other influential factors and the incidence of bad split osteotomies in Dal Pont and Hunsuck techniques.

## CONCLUSION

The duration of osteotomy and splitting is much shorter when the Hunsuck technique is employed, and the incidence of unfavorable fractures is also less compared to the Dal Pont osteotomy technique. Further studies are necessary for stronger relevancy.

## FUNDING

None. This study was self-funded

## CONFLICTS OF INTEREST

The authors have no conflicts of interest to disclose. 
